# Cases of Fractures

**Published:** 1857-02

**Authors:** Frank Hastings Hamilton

**Affiliations:** Buffalo, N. Y.


### BUFFALO MEDICAL JOURNAL

AND

### MONTHLY REVIEW.

VOL. 12. FEBRUARY, 1857. NO. 9.

### ORIGINAL COMMUNICATIONS.

ART. I. — *Cases of Fractures.* Reported by Frank Hastings Hamilton, M. D., Buffalo, N. Y.

(Continued from Page 459 )

### FIBULA.

(I have no cases of fracture of the upper third to report.)

### MIDDLE THIRD.

Case I. — *Simple fracture, with dislocation of tibia inward. Result imperfect.*

J. L. B., of Utica, æt. 12; by the kick of a horse his left tibia was dislocated inward, and the fibula broken five inches above its lower end. Dr. Ford, of Cazenovia, reduced the bones.

After two years, the tibia remains slightly displaced inward, and the fibula rests against the tibia at the seat of fracture. The motions of the limb are unimpaired, and its usefulness is in no way diminished.

CASE II.— *Compound fracture, with dislocation of tibia inward. Result nearly perfect.*

Robert Campbell, æt. 20, fractured the fibula of his left leg near its middle. Fracture compound, and complicated with an inward dislocation of the tibia. Dr. Wilson, of Providence, R. I., was employed.

Five years after the injury was received, I found the limb sound, and nearly perfect.

CASE III.— *Compound fracture, with dislocation of the tibia inward. Result perfect.*

Harrington Whiton, æt. 14, fractured the left fibula near its middle. The tibia was at the same time partially dislocated inward.

Twenty-eight years after, I could not discover any trace of the injury.

LOWER THIRD.

CASE IV.— *Simple fracture. Result perfect.*

E. T. Bacchus, æt. 40, broke left fibula, near the ankle, Dec. 7th, 1836. Mr. B. is a farmer, and was, at the time of the accident, in excellent health. He was walking upon an icy sidewalk, when his foot slipped, and he fell, breaking his leg by the fall.

I applied within an hour, Dupuytren's splint along the inside of his leg, and sent him home.

Six months after, his limb was sound and without deformity.

CASE V.— *Simple fracture. Result perfect.*

Robert Purcell, of Attica, æt. 57, fell from a scaffolding and fractured the fibula about two inches above the ankle. Dr. A. P. Curtis, of Attica, in attendance. Active inflammation followed.

Four weeks after the accident he called upon me, and I found the bone firmly united. I could not at this time detect any deformity, but Dr. Curtis has since informed me that he thinks a slight deformity ensued.

This gentleman has had one of his legs broken twice before, and in each instance the fractures were treated by Dr. Curtis, and united without deformity.

CASE VI. — *Simple fracture. Result perfect.*

Charles Billings, of Buffalo, æt. 34, fractured the left fibula about three inches above its lower end. It was treated by Dr. Chapin, of Buffalo, N. Y.

Fourteen years after the accident the limb was perfect.

CASE VII. — *Simple fracture. Result perfect.*

Isaac Stafford, æt. 34, broke the left fibula in its lower third. Dr. Low, of Guilderland, N. Y., dressed the limb.

Twenty-four years after the accident I could not detect any traces of the fracture.

CASE VIII. — *Simple fracture. Result perfect.*

Frank Whalin, æt. 26, fell from a scaffolding, July 29th, 1853, breaking the right fibula three inches above the ankle. I applied Dupuytren's splint, secured with a starch bandage.

He died in May, 1854, and at this time the limb seemed perfect. He had walked for many months without any appearance of halt or lameness.

CASE IX. — *Simple fracture. Result perfect.*

Michael Schenlin, of Buffalo, æt. 25. Jan. 20, 1855, a heavy gate fell upon the outside of his left leg, breaking the fibula three and a half inches above the ankle. No surgeon was called until the 23d, three days after the accident. The limb was then much swollen. I could not feel the fibula sufficiently to determine whether the fragments were displaced, but crepitus was distinct.

I directed no applications except cold water irrigations. On the seventh day the swelling had subsided. Fragments in line, but not united.

This patient recovered in about six weeks, without either lameness or deformity.

CASE X. — *Simple fracture; with partial dislocation of tibia inward. Result perfect.*

Mrs. Catharine Jordan, of Buffalo, aged 60 years. Mrs. J. fell on the sidewalk, producing a partial dislocation inward of the left tibia, and a fracture of the fibula.

The fracture of the fibula could not be felt, nor could I detect crepitus, but its existence was assumed from the great degree of flexibility about the joint.

The tibia was easily restored to place, and the leg was firm and apparently perfect two months after the injury was received.

CASE XI.—*Simple fracture. Result imperfect.*

James Shepard, æt. 20, fractured the fibula of his right leg, about two inches above its lower end.

Dr. John McBeth, of Erie Co., was employed.

Four years after, I found the foot slightly turned out, and there remained considerable bony enlargement around the seat of fracture. The fibula had united bent inward toward the tibia. The motions of the joint were free and perfect.

CASE XII.—*Simple fracture. Result imperfect.*

John Sanford, of Schenectady, æt. 32, fractured his left fibula about two inches above its lower end. Dr. Day, of Schenectady, was employed.

I examined the limb twenty years after, and found some deformity remaining. It occasionally swells and becomes painful.

CASE XIII.—*Simple fracture, with dislocation of the tibia inward. Result nearly perfect.*

Charles Sauer, of Buffalo, aged about 30 years; while carrying a weight upon his shoulders, slipped upon the sidewalk and fell, dislocating the left ankle, and fracturing the fibula four inches from its lower end. I was in attendance soon after the accident occurred, and reduced the dislocation by very moderate extension, made with my hands. The leg was flexed upon the thigh, while extension was made at the foot. We applied Dupuytren's splint. Only moderate inflammation followed, and the limb was free from all anchylosis six weeks after the injury was received.

About two years after, Aug. 2, 1856, I examined the limb and found that the ankle joint remained larger than the other, and that occasionally it became considerably swollen and somewhat painful. The malleolus internus does not project at all unnaturally, nor does the foot turn out. The fibula is united in perfect line, the fragments not inclining in either direction.

Case XIV. — *Simple fracture, with dislocation of the tibia inward. Result imperfect.*

Jonathan Robinson, æt. 32, dislocated the lower end of the tibia inward, breaking the fibula about two or three inches above the ankle.

Dr. Hosmer, of Lancaster, N. Y., reduced the dislocation and treated the fracture.

I examined the leg twelve weeks after, and found the tibia remaining slightly displaced inward, and the fibula bent inward, toward the tibia, at the seat of fracture. The ankle was much swollen.

Case XV. — *Simple fracture, with dislocation of tibia inward. Result imperfect.*

—— Terry, of Mount Morris, aged about 35 years. The accident occurred in stepping forward from a canal boat upon an irregular surface.

Dr. Shuler, of Lockport, immediately reduced the bones and applied Dupuytren's splint. He subsequently came under my care in Buffalo. The inflammation consequent upon the injury, soon made it necessary to remove the splint. He left me at the end of five weeks with the fracture united, and the swelling nearly dispersed, but with considerable stiffness in the joint, and with some slight projection of the malleolus internus.

Case XVI. — *Simple fracture, and inward displacement of tibia. Result imperfect.*

Philip Carey, of Ireland, æt. 22, fell with his left leg under him, and in this situation another man fell upon him, breaking the fibula of this leg about three or four inches above the ankle-joint, and displacing the tibia inward.

An Irish bonesetter reduced the dislocation, and treated the fracture. No splints were applied. He was confined to his bed six weeks.

Twenty years after this, while he was an inmate of the Buffalo Hospital, March 30, 1855, I examined the leg.

The fibula is united with the ends of the fragments displaced; the lower end of the upper fragment being behind the upper end of the lower. The motions of the ankle-joint are not free, and it is occasionally painful when too much exercised.

CASE XVII.—*Simple fracture of external malleolus, with partial inward displacement of the tibia. Result imperfect.*

Abel Godard, of Dansville, æt. 21, partially dislocated the tibia inward, fracturing also the malleolus externus. Dr. Reynale, of Dansville, N. Y., was employed.

Eight months after the accident occurred, I found the tibia remaining slightly displaced, and the lower fragment of the fibula carried downward.

CASE XVIII.—*Simple fracture, with partial outward displacement of tibia. Result imperfect.*

L. I. Barrows, of Utica, æt. 12, was kicked by a horse, breaking the radius of the left arm, and also at the same time the left fibula, four or five inches above the ankle. The tibia was also slightly displaced outward.

Ten years after the accident, while he was a student of medicine, I examined the limb. The tibia still remained somewhat displaced outward, and the fibula was bent in against the tibia at the seat of fracture. The functions of the limb were unimpaired.

Dr. Ford, of Cazenovia, was his surgical attendant.

CASE XIX.—*Comminuted fracture, complicated with a compound inward dislocation of tibia. Amputation.*

Aug. 12, 1853, Mrs. Abner Birmingham, of Buffalo, aged about 25 years, was caught under a mass of falling boards, breaking her right leg and right arm.

Drs. Nott and Boardman, with myself, were called. The right fibula was broken through the lower third into several fragments, while the tibia was thrust inward and downward, penetrating the skin to the extent of two or three inches, and removing from the end of the bone both periosteum and cartilage. The bleeding, from several arteries and veins, was quite profuse; she was suffering great pain.

Several hours had now elapsed since the accident, and I proceeded immediately to amputate the leg, by candle light, at a point somewhat below the middle.

Her recovery was slow, but complete.

CASE XX.— *Compound fracture, complicated with partial inward displacement of the tibia, with dislocation of the astragalus, fracture of the femur, and other injuries. Death on the tenth day.*

Michael O'Shea. (See Case CIX of Fractures of Femur.)

CASE XXI.— *Simple fracture of the fibula, complicated with a compound, consecutive dislocation of the tibia forward. Result imperfect.*

Michael McLaughlin, aged 35 years, of Buffalo. A heavy bar of iron fell upon McLaughlin's instep, resulting in violent inflammation and a sloughing of all the structures in front of the ankle-joint. The fibula was broken near its lower end by the same accident.

Dr. Pratt, of Buffalo, was employed, and treated the case with light splints, but with his attention directed chiefly to the prevention of inflammation. Fifteen days after the injury was received I saw the patient with Dr. Pratt. No displacement of the bone had yet occurred. Subsequently he became an inmate of the hospital and remained under my care until he was dismissed.

When the slough separated, the tibia began gradually to abandon its socket and to slide forward, and this notwithstanding our constant efforts to retain it in place. A small piece of the front and lower end of the tibia at length exfoliated, and from this time the closure of the wound advanced rapidly. We were now able by the careful application of dressings with compresses, to gradually return the bone to its place, nd when the wound had completely healed, the tibia was again restored to position. About six months elapsed before the cure was complete. No deformity then remained, but the ankle was partially anchylosed.

(To be Continued.)

